# Label-Free Sensors Based on Graphene Field-Effect Transistors for the Detection of Human Chorionic Gonadotropin Cancer Risk Biomarker

**DOI:** 10.3390/diagnostics8010005

**Published:** 2018-01-08

**Authors:** Carrie Haslam, Samar Damiati, Toby Whitley, Paul Davey, Emmanuel Ifeachor, Shakil A. Awan

**Affiliations:** 1 Wolfson Nanomaterials and Devices Laboratory, School of Computing, Electronics and Mathematics, Faculty of Science and Engineering, University of Plymouth, Plymouth PL4 8AA, UK; carrie.haslam@plymouth.ac.uk (C.H.); toby.whitley@plymouth.ac.uk (T.W.); paul.davey@plymouth.ac.uk (P.D.) emmanuel.ifeachor@plymouth.ac.uk (E.I.); 2 Department of Biochemistry, Faculty of Science, King Abdulaziz University (KAU), Jeddah 21589, Saudi Arabia; sdamiati@kau.edu.sa

**Keywords:** graphene, electrochemical biosensors, cancer, diagnosis, electrical detection, Alzheimer’s disease, dementia, neurodegenerative disorders, cardiovascular, blood biomarkers, antibodies, proteins

## Abstract

We report on the development of label-free chemical vapour deposition (CVD) graphene field effect transistor (GFET) immunosensors for the sensitive detection of Human Chorionic Gonadotropin (hCG), a glycoprotein risk biomarker of certain cancers. The GFET sensors were fabricated on Si/SiO_2_ substrate using photolithography with evaporated chromium and sputtered gold contacts. GFET channels were functionalised with a linker molecule to an immobile anti-hCG antibody on the surface of graphene. The binding reaction of the antibody with varying concentration levels of hCG antigen demonstrated the limit of detection of the GFET sensors to be below 1 pg/mL using four-probe electrical measurements. We also show that annealing can significantly improve the carrier transport properties of GFETs and shift the Dirac point (Fermi level) with reduced p-doping in back-gated measurements. The developed GFET biosensors are generic and could find applications in a broad range of medical diagnostics in addition to cancer, such as neurodegenerative (Alzheimer’s and Parkinson’s) and cardiovascular disorders.

## 1. Introduction

Since the discovery and isolation of graphene from graphite in 2004, the two-dimensional material has shown potential to revolutionise many technological areas owing to its remarkable electrical, optical, mechanical and thermal properties [1,2,3]. Graphene is a mono-atomic, planar network of sp^2^-bonded carbon atoms arranged in a honeycomb lattice. In particular, its chemical stability, low electrical noise, significant surface-to-volume ratio, biocompatibility and field-effect make graphene potentially an ideal platform for a variety of biosensing applications [4]. The extraordinary surface-to-volume ratio of graphene facilitates a high density of disease specific capture molecules or antibodies to be chemically immobilised onto its surface via covalent or non-covalent functionalisation techniques. A key benefit of the latter is that the electrical transport properties of graphene are not degraded as the mobility and carrier properties are maintained. This can enhance the sensitivity of the biosensor and thereby improve its limit of detection (LoD). Currently, a number of biosensor techniques are being explored to detect the presence of a given biomarker, such as electrical, optical, resonant, acoustical, quartz crystal microbalance and surface plasmon resonance (SPR) [5]. Whilst each technique has its advantages and disadvantages, the electrical technique we are exploring offers some key benefits that make it a prime candidate for eventual point-of-care (PoC) applications in the detection of specific cancer biomarkers and early diagnosis. These include label-free detection, high sensitivity, multiplexing, low-cost and integration with a range of existing electronic systems. In contrast with our field-effect transistor (FET) biosensors, there are also other electrical techniques, such as cyclic voltammetry (CV), impedimetry and ampereometry functioning to detect a variation in resistance and/or capacitance, that may be comparable to the FET approach in terms of sensitivity. The developed FETs will be generic sensors and are therefore expected to have numerous other applications ranging from neurodegenerative disorders (ND), such as Alzheimer’s [6] and other dementias, and cardiovascular to infectious diseases and environmental monitoring.

Here we report on the chemical vapour deposition (CVD) graphene field-effect transistor (GFET) biosensors aimed at detecting the presence of Human Chorionic Gonadotropin (hCG) protein biomarkers in samples from patients at different stages of disease progression. The proof-of-concept GFETs were functionalised with monoclonal anti-hCG antibodies for the detection of hCG protein biomarkers, label-free. hCG is a glycoprotein hormone (37 kDa) and its concentration level serves as a clinical marker associated with testicular, pancreatic and prostate cancer [7,8,9], as well as pregnancy. As with many diseases, early cancer recognition and diagnosis can be critical for treatment and management to be effective, as well as enabling optimum health care provision and potentially opening novel routes to be investigated to prevent the development of the disease [10,11,12]. There is therefore an urgent need to develop diagnostic sensors and analytical tools for the accurate identification and early diagnosis of cancer. hCG is composed of two subunits, α and β, which are non-covalently linked by hydrophobic and ionic interactions. The α subunit is known to be common and the β subunit is unique to hCG, conferring biological activity to the hormone. The β subunit is composed of 145 amino acids [13,14,15,16,17]. As a pregnancy biomarker hCG is usually secreted by the trophoblast cells of the placenta peaking at ~10 weeks within the human gestation period. The chief function of hCG is considered to maintain the corpus luteum and promote the production of progesterone during pregnancy by the ovarian granulosa cells, whilst endorsing foetal growth [18,19,20,21,22]. A typical, commercial biosensor that is designed to detect hCG and establish pregnancy exhibits sensitively down to a nano-molar range [23]. It has been previously estimated that 95% of pregnancies can be detected at a sensitivity of 1.24 µg/mL at the time of an expected menstrual period [24,25]. Our GFET biosensors detailed here exhibit a significantly lower limit of detection of ~0.1 pg/mL (determined using a 4-probe electrical technique) compared to a commercial biosensor designed specifically for hCG detection. There are number of other studies that have investigated antibody–antigen biomarker detection to analyse the necessary LoD of their sensors, e.g., Heideman et al. [26], who developed a Mach–Zehnder interferometer (MZI) immunosensor, based on a silicon substrate with a detection limit of 50 pM [26,27] and Mao et al., who detected immunoglobulin G (IgG) at the 2 ng/mL level [28]. In respect to hCG detection via graphene, Teixeira et al. [15] applied chemically-modified multi-layer epitaxial graphene to serve as the sense electrode (functionalised with APTES). The device displayed a LoD of 0.62 ng/mL [15]. In addition, Teixeira et al. produced an immunosensor based on screen-printed electrodes (SPEs), with graphene functioning as the working electrode to detect hCG. The LoD was determined to be 0.286 pg/mL [29]. In addition, Islam et al. reported a hCG detection limit of ~1 pg/mL using a CVD graphene biosensor [30]. The developed GFET biosensors will also need to be validated against the gold standard in the field, that of immunoassay enzyme-linked immunosorbent assay (ELISA) for both sensitivity and specificity. In addition, since performing ELISA tests can be time consuming and requires a high level of operator expertise and complex equipment, making it expensive, we envisage that GFET biosensors could present a potential low-cost, fast, accurate and reliable alternative technology platform.

## 2. Materials and Methods

### 2.1. Materials

Monolayer Graphene on a Si/SiO_2_ wafer was purchased from Graphenea (San Sebastián, Spain). The required chemicals applied for GFET fabrication were photoresist (PR), lift-off resist (LoR), Microposit developer and Microposit remover, all purchased from A-Gas Electronic Materials (Warwickshire, UK).

The hCG antigen and complementary anti-hCG monoclonal antibody were purchased from Abcam (Cambridge, UK). The stocks were stored at −20 °C. Linker molecule 1-pyrenebutanoic acid succinimidyl ester (Pyr-NHS), bovine serum albumin (BSA) blocking solution andPhosphate-Buffered Saline (PBS) tablets were purchased from Sigma Aldrich (Dorset, UK).

Electrical characterisation and analysis were implemented under ambient conditions via a Keysight B1500A semiconductor device parameter analyser interfaced to aMPS150 probe station (Cascade Microtech, Thiendorf, Germany)*.* Current–voltage measurements (I_D_–V_D_ and I_D_–V_G_) were acquired as a function of gate voltage (V_G_) from −100 V to +100 V with I_D_–V_D_ curves from −100 mV to +100 mV. The I_D_–V_D_ and I_D_–V_G_ curves were measured for each separate functionalisation stage.

Graphene characterisation was performed using an XPLORA Raman spectroscopy system (HORIBA, Northampton, UK). The XPLORA system operated at a wavelength of 532 nm, with ~4 mW of incident power. The XPLORA Raman system was combined with an OLYMPUS BX41 microscope (Shinjuku, Japan).

To define and develop the necessary graphene channels and metallic contacts of Chromium (Cr) and Gold (Au), the Mask aligner J500/VIS (OAI Optical Associates Inc., San Jose, CA, USA) was deployed. A conventional fan oven (WTB Binder, Tuttlingen, Germany) was employed to perform necessary annealing processes, as well as for pre- and post-baking stages during the photolithography processes. The Hotplate SH8 (STUART, Stone, UK,) and Ultrasonic Cleaner (UPCORP, Freeport, IL, USA) were used for pre- and post-baking procedures and PR removal, respectively. An Argon (Ar) plasma etching technique was used for graphene channel formation by means of a three-target, 6-inch sputtering machine (Nordiko Limited, Havant, UK). To aid in the formation of robust, metallic electrodes, an Edwards Thermal Evaporator was deployed for the evaporation of Cr.

### 2.2. Fabrication of CVD Graphene FETs

The GFETs were fabricated using high quality CVD graphene to form transducer channels on Si/SiO_2_ substrate. A conventional photolithographic patterning and metal lift-off technique was used to form the necessary graphene channels, as well as the source, drain and voltage sense electrodes. The fabrication process involved spin-coating the CVD graphene samples with Lift-off Resist (LoR) at 3000 revolutions-per-minute (RPM) for a few seconds. The samples were then placed in a fan oven for pre-baking. The purpose of the pre-bake was to densify the LoR to assist with the formation of clear, graphene channels. Subsequently, the samples were dried at ambient temperature. Next, the samples were spin-coated with a layer of positive Photoresist (PR). Immediately after PR deposition, the samples were post-baked on a hotplate. The hotplate solidifies the PR and removes any solvents remaining on the samples. This process generated ~500 nm of PR film on the surface of graphene. Successively, the coated samples were positioned in a mask aligner and exposed to ultra-violet (UV) radiation for lithographic patterning of graphene channels. The UV exposed samples were also submerged in a chemical developer before being placed in a vacuum chamber to dry. Alternatively, the samples can be left to dry at ambient temperature, outside of the vacuum chamber. An additional post-baking stage was applied to the samples following the drying and placed on a hotplate under an intermittent deep ultraviolet source (DUV). This exposure to DUV can reduce the quantity of PR/PMMA residue on the substrate surface prior to plasma etching, whilst reducing contact resistance.

The chemically developed and dried samples were transferred into a sputtering machine and exposed to Ar plasma etching for a few minutes. Throughout the process, the inert Ar gas functions as the gaseous etchant and removes the exposed regions of graphene, whilst leaving behind the shaped graphene channels (previously coated and protected with PR). Graphene shaping is effectively complete after an etching time of a few minutes at high power. The sputtering machine was also used to sputter and form the associated metallic electrodes, 30 nm of Au. Following successful shaping, the samples were transferred to a chemical remover to dissolve the LoR and PR remaining on the substrate to expose the graphene channels. The samples were immersed in the chemical remover, before being placed in an ultrasonic bath for ~2 h at ~60 °C. The samples were subsequently rinsed and bathed in de-ionised water and transferred into a vacuum chamber to dry for few hours.

For the final stage of GFET fabrication, metallic Cr and Au electrodes were formed on the graphene channels. The samples were post-baked in a fan oven, instead of being irradiated with DUV. This post-baking stage is necessary to remove the solvents located within the PR and eliminate any additional water. Following photolithography, thermal evaporation of Cr was performed using an Edwards Thermal Evaporator for a few seconds. The Cr functions as an adhesive layer between graphene and the Au metallic contacts. The Cr target material was heated to ~2000 °C and evaporated within a high vacuum. The heated coil of Cr is applied to form a thin film of ~5 nm on the surface of the graphene samples. The metallic contacts are fully formed after sputtering 30 nm of Au on the Cr film. After the Au sputtering process, the initial chemical remover step is repeated and the final samples are dried in a vacuum chamber for a few hours, prior to electrical characterisation.

**Figure 1:**
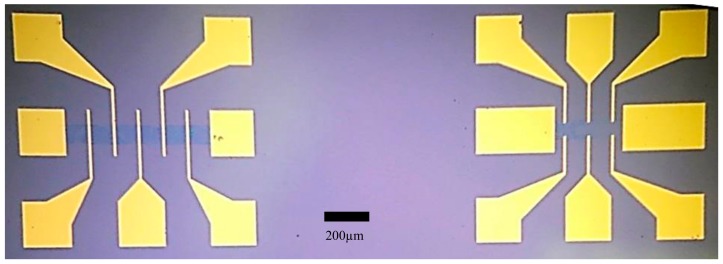
Graphene field effect transistor (FET) devices with asymmetric (**left**) and symmetric (**right**) voltage sense electrodes. Graphene channels, in the central regions of the FET devices, have a dark blue contrast compared to the Si/SiO_2_ substrate.

For this study, a number of CVD graphene samples were fabricated. Each sample consisted of fifteen GFET devices (with an area of ~1 cm^2^) in total, i.e., five GFET devices consisted of five asymmetric Cr/Au voltage sense electrodes with an associated graphene channel length of 720 μm, whereas the remaining ten GFET devices consisted of six symmetric voltage sense electrodes with a channel length of 300 μm, as shown in Figure 1. The width of all GFET channels was fixed at 80 μm.

## 3. Results

### 3.1. Characterisation of CVD Graphene Using Raman Spectroscopy

**Figure 2:**
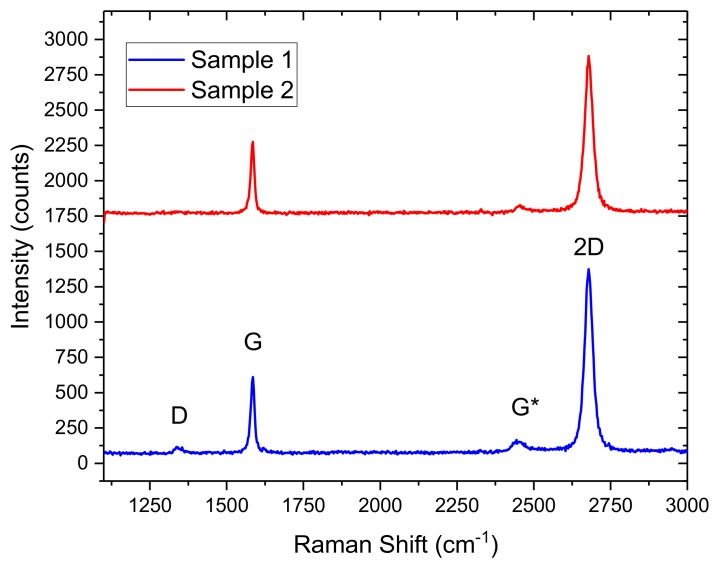
Raman Spectra of quality and defected chemical vapour deposition (CVD) graphene before being deployed as the transducer element in a graphene field effect transistor (GFET) biosensor. The defected sample (blue) shows a prominent D-band.

Raman spectroscopy was performed on two representative CVD samples to analyse the quality of graphene and to estimate various properties, such as number of layers [31], doping and residues from the fabrication process. Random spot Raman measurements were carried out at the central region of bare samples. The Raman spectra were measured with a 10× objective, under ambient conditions with an incident power of ~4 mW and a wavelength of 532 nm. Figure 2 shows Raman spectra from the two samples with an intensity ratio of the 2D peak to G peak of I(2D)/I(G) ~2 and one sample shows a significant D peak, whereas it is absent in the other. The intensity ratio I(2D)/I(G), position and full width half maximum (FWHM) of the G and 2D peaks confirm the presence of monolayer graphene. The presence and intensity of the D peak in one of the samples also indicates that the graphene in that sample contains defects. Raman mapping of a few regions of the graphene wafer (before it was diced into 1 cm^2^ samples) confirmed that the majority of the regions consisted of mono-layer graphene.

### 3.2. Electrical Characterisation of GFETs

**Figure 3:**
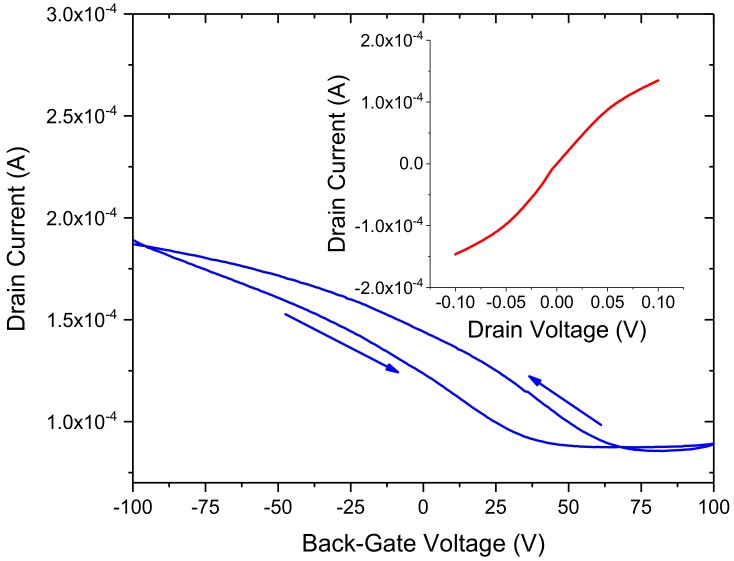
I_D_–V_G_ at V_D_ = 100 mV and the corresponding I_D_–V_D_ (inset) of a CVD graphene FET sensor. The arrows indicate forward (−100 V to 100 V) and reverse (100 V to −100 V) sweep of the back-gate voltage showing hysteretic behavior.

The GFETs were characterised using 2-probe, 4-probe and back-gated measurements under ambient conditions. The bare GFETs (i.e., without any biomolecule functionalisation) were first measured to extract the I_D_–V_D_ characteristics of the fifteen devices on each 1 cm^2^ sample. A comparison between 2-probe, 4-probe and back-gated data enabled us to extract the contact resistance, mobility and sheet resistance of the graphene channels within the GFET devices. A typical 4-probe I_D_–V_D_ and I_D_–V_G_ measurement from a GFET sensor, using a Keysight parameter analyser interfaced to a Cascade probe station, is shown in Figure 3. The I_D_–V_D_ data shows approximate linear response over the ±60 mV range and a slight non-linearity beyond this range probably due to Schottky contacts or charge traps at the interface with the Si/SiO_2_ substrate [32]. This is further confirmed from the I_D_–V_G_ data which shows a characteristic hysteretic behavior due to the presence of charge traps at the interface. The Dirac point is found to vary from 50 to 80 V depending on the voltage sweep and its positive magnitude indicates that the graphene channel is p-doped (at V_D_ = 100 mV). The 4-probe resistance of the graphene channel is found to be ~700 Ω (with gate potential at 0 V), whereas the mobility is approximately 1200 cm^2^/Vs.

**Figure 4:**
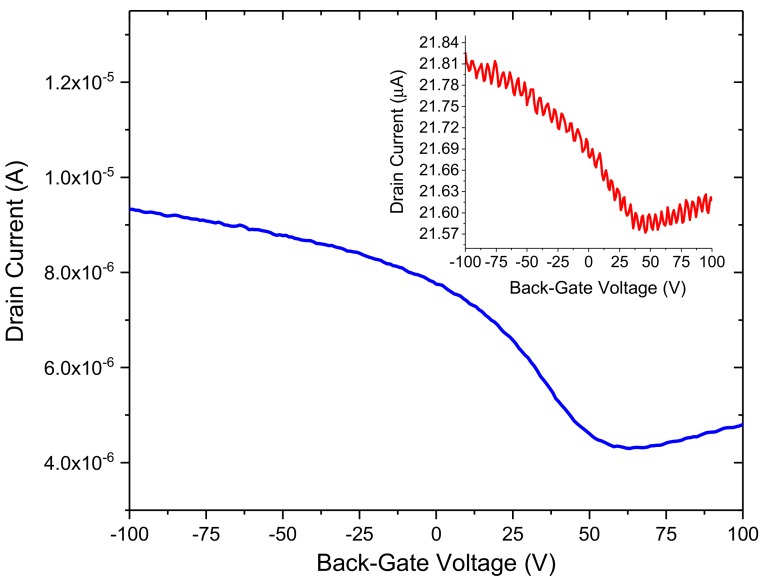
Annealed I_D_–V_G_ curve of a CVD graphene FET and its corresponding I_D_–V_G_ (inset) before annealing, both at V_D_ = 100 mV.

We have also explored the effect of annealing on our devices in order to improve the overall performance of the bare GFET sensors. Figure 4 displays the results from one device before and after annealing. Before annealing, the device showed a relatively low conduction level and its Dirac point was in excess of 40 V. However, after annealing the sample, the Dirac point could now be discerned and shifted to approximately 23 V (with p-type doping). This is likely due to desorption of oxygen and water molecules from the surface of graphene [33,34,35]. In pristine graphene, the Dirac point would ideally be located at zero gate voltage. A Dirac point situated relatively far from the zero point indicates unintentional doping, which could be due to the fabrication process. The location of the Dirac point is dependent on the difference between the work function of the gate, metallic contacts, and the graphene channel, as well as doping and the presence of biomolecules adsorbed on the surface [36]. From the knowledge of the Dirac point and shape of the I_D_–V_G_ curve near its vicinity, a number of properties of the channel can be estimated, such as mobility, doping, defects etc.

### 3.3. Functionalisation of GFETs

To facilitate antibody (Ab) immobilisation, we used a linker molecule Pyr-NHS ester. The linker retains a succinimidyl ester group, which interacts with the amino groups on the anti-hCG Ab surface to generate a stable, amide bond. In addition, the Pyr-NHS has an aromatic pyrenyl group, which can strongly interact with the graphene surface, via a non-covalent π–π stacking. Non-covalent binding is dependent on physical adsorption of the biomolecule, using hydrophobic, hydrophilic, electrostatic and van der Waals interactions. Thus, Pyr-NHS ester causes the pyrene group at one end of the linker to strongly bind to the graphene surface via π–π interactions and the succinimidyl ester group will covalently react with the amino group (NH_2)_ of the Ab without requiring a high pH [37]. The non-covalent attachment of Pyr-NHS ester with graphene does not disrupt the electrical properties of graphene, whereas covalent bonding can lead to scattering and the introduction of impurities in the graphene sp^2^ lattice. This type of immobilisation typically leads to a self-assembled monolayer (SAM) of molecules on the surface of graphene, although the Abs may remain randomly orientated.

The GFET devices were biologically modified and converted into biosensors via the drop casting of biomolecules. Initially, Pyr-NHS ester linker molecules were applied to cover the surface of the graphene channels at a concentration of 2 mM. The samples were incubated at low temperature for a few hours before rinsing with PBS and drying at ambient temperature. Once the samples were dry, electrical characterisation was performed. Following attachment of Pyr-NHS ester and electrical characterisation, anti-hCG Ab was applied to the samples and again incubated before rinsing with PBS and drying at ambient temperature. The same process was repeated for BSA deposition. BSA was applied to block additional sites between the Ab regions which prevents non-specific binding.

**Figure 5:**
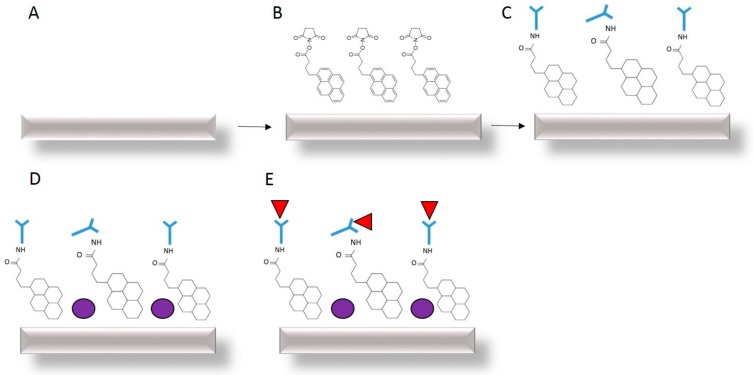
GFET functionalisation and measurement of (**A**) bare graphene (**B**) non-covalent attachment of Pyr-NHS ester linkers with graphene (**C**) anti-human chorionic gonadotropin (hCG) antibody (light blue) attachment to the linkers (**D**) BSA blocking (dark blue) and (**E**) detection of hCG antigen (red) binding with the antibody. Each stage is characterised using 4-probe electrical measurements of I_D_–V_D_ and I_D_–V_G_.

Finally, hCG antigen was applied to the samples at a concentration ranging from 0.1 pg/mL to 1 ng/mL and characterised using 4-probe electrical measurements. Samples were then incubated in ambient conditions for 1 h, prior to a PBS rinse. Figure 5 illustrates the steps involved within the functionalisation process.

### 3.4. Atomic Force Microscopy of Functionalised GFETs

**Figure 6:**
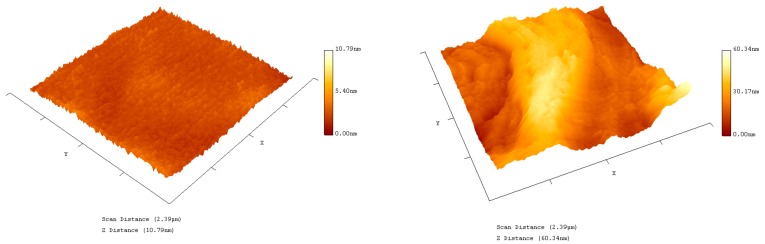
Atomic Force Microscopy (AFM) image of bare graphene on Si/SiO_2_ (**left**) and functionalised with Pyr-NHS linker, Ab, BSA and the corresponding hCG antigen (**right**).

Further characterisation of the functionalised GFETs was performed using Atomic Force Microscopy (AFM, Pacific Nanotechnology, Santa Clara, CA, USA) and probe tips from Nano World, Neuchâtel, Switzerland, Multiple AFM images were generated in respect to the various necessary functionalisation steps. The difference in height and morphology at the hCG functionalisation stage is displayed in Figure 6. The AFM image illustrates the successful immobilisation of Pyr-NHS linker, Ab, BSA and the corresponding hCG antigen molecules on the surface of the graphene FETs. The hCG antigen is measured to be ~7–10 nm in height.

### 3.5. Scanning Electron Microscopy of Functionalised GFETs

**Figure 7:**
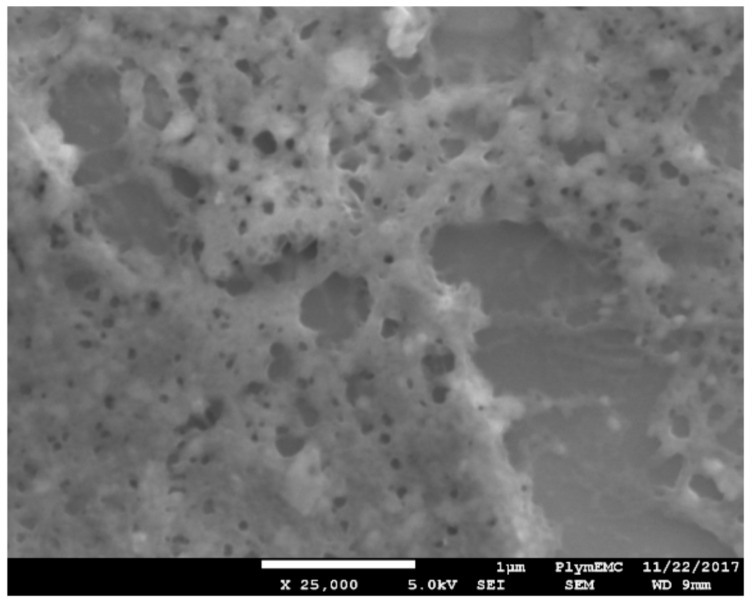
SEM image displaying bare graphene functionalised with Pyr-NHS linker, Ab, BSA and the corresponding hCG antigen. Scale bar is 1 µm.

An SEM image (using a JOEL JSM-7001F) in Figure 7 shows a GFET device with 10 pg/mL of hCG antigen on the surface of graphene, including immobilisation of Pyr-NHS ester linker and functionalised with Ab and BSA blocking molecules. The image (taken at 25,000 times magnification) shows hCG appearance to be foam like with relatively large voids over the 5 µm × 5 µm region. The hCG appears also not to be uniformly distributed, indicating that further optimisation may be needed to achieve a uniform distribution of the antigen on the surface of graphene to enable consistent and repeatable response of the GFET biosensors.

### 3.6. Detection of hCG Concentration

**Figure 8:**
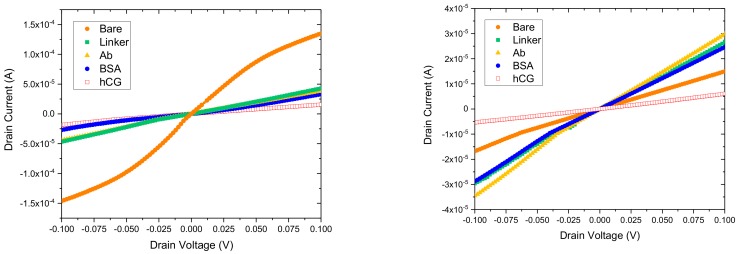
Characteristic I_D_–V_D_ curves from the GFET sensors at different stages of biomolecule exposure for 1 pg/mL (**left**) and 1 ng/mL (**right**) hCG concentration levels.

**Figure 9:**
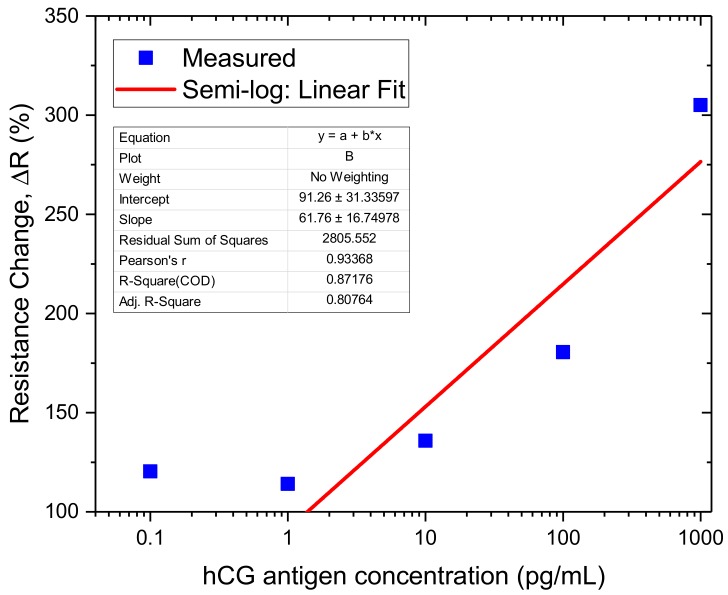
Measured resistance change coefficient with hCG concentration and a least-squares linear fit (on a semi-log plot) to the data from the 1 pg/mL to 1 ng/mL range.

The fabricated GFET sensors were characterised using 4-probe electrical measurements at each stage of the functionalisation and binding interactions of the anti-hCG antibody with hCG antigen at varying concentration levels of the latter. All measurements were performed in ambient conditions. Figure 8 shows the response curves of two GFET biosensors when exposed to 1 pg/mL and 1 ng/mL hCG antigen. The corresponding changes in resistance at each stage and concentration level are shown in Table 1. A striking feature of the results shown in Figure 8 and Table 1 is that whilst the change in resistance coefficient ΔR for 1 pg/mL is positive for all stages of the functionalisation, this is not the case for the 1 ng/mL concentration level. In this case, the coefficient is negative until the antibody binding stage but then becomes positive. This may be related to the complex interplay between the potential distribution in the graphene channel, attached molecules and the interface with the substrate. In addition, despite 3 orders-of-magnitude difference between the 1 pg/mL and 1 ng/mL concentration levels, the change in resistance coefficient is ~114.1% and ~305%, respectively. In contrast, we measured ΔR ~ 135.9% for 10 pg/mL, ΔR ~ 180.4% for 100 pg/mL and a LoD of the sensors to be ~0.1 pg/mL, as shown in Figure 9. The response of the GFET sensors to hCG antigen concentration is found to be logorithmic (linear fit on a semi-log plot) over the 1 pg/mL to 1 ng/mL range, as shown in Figure 9. The least-square linear fit to the measured GFET 4-probe resistance change shows reasonably good fit with R^2^ ~ 0.8. The corresponding sensitivity of the GFET biosensors is found to be ~(61.8 ± 16.7)%/decade of hCG concentration over the 1 pg/mL to 1 ng/mL range. This represents a significant sensitivity level for hCG antigen label-free detection. However, it is worth highlighting that our GFET biosensors have not yet been optimised for numerous parameters (mobility etc.) to achieve even higher sensitivity levels and lower detection limits. These aspects are currently being explored and will be reported on in subsequent future publications. 

The data in Table 1 for the measured 4-probe resistance of the GFET sensors at 1 pg/mL and 1 ng/mL concentration levels also includes the change in resistance coefficient ΔR. This is calculated relative to the two nearest stages, i.e., bare graphene to linker, linker to Ab, etc. The physical mechanism for this change in resistance of the graphene channel at each functionalisation stage is due to the attached molecules either donating or accepting electrons to/from the channel. Thus, sensitivity of the sensors can be estimated from ΔR for hCG (or change in resistance from BSA to the hCG analyte).

## 4. Discussion

The CVD graphene FET biosensors have been characterised at all stages of the fabrication process (Raman, AFM, electrical) and functionalisation (Raman, AFM, SEM, electrical) to establish a robust and reproducible process for the development of the proof-of-concept sensors. Our data presented in this study has demonstrated that CVD graphene FETs can sensitively detect hCG antigen with approximately three to four orders of magnitude lower concentration level compared to commercially available sensors. In addition, the GFET sensors can be operated in a resistive or back-gated mode and in the future we aim to compare the sensitivity of these two approaches. However, the data in Figure 8 shows clear characteristic curves for a label-free resistive GFET sensor which we also observed for the 10 pg/mL and 100 pg/mL concentration levels of the hCG antigen. As shown in Figure 3, back-gated GFET sensor properties can be influenced due to the presence of water molecules on the surface of graphene, charge traps at the interface between graphene and the supporting substrate (in our case Si/SiO_2_) leading to hysteretic effects. Therefore, a robust comparison of the resistive with back-gated GFET sensors will require careful device design and optimisation. 

Annealing of the GFET biosensors showed remarkable improvements in their electrical performance, in terms of enhanced carrier transport properties and Dirac point shift due to oxygen and water molecules being desorbed from the surface of graphene. Work is currently underway to compare annealed devices with those that have been fabricated in the same batch but not annealed. In addition, all the results reported in this study are based on direct current (DC) 4-probe resistance measurements. We therefore anticipate that further improvements in S/N can also be achieved using alternating current (AC) or radio-frequency (RF) detection techniques [38,39].

## 5. Conclusions

We have demonstrated the use of CVD graphene FETs on Si/SiO_2_ substrate as sensitive label-free immunosensors for the proof-of-concept detection of hCG antigen, with a LoD below ~1 pg/mL. Although characteristic current-voltage response curves of the GFETs have been established, the sign of the resistance change coefficient ΔR has a strong dependence on the potential environment of the graphene channels. In addition, we have also shown that annealing the GFET sensors can significantly improve their performance and potentially lead to even more sensitive devices. In the near future, the GFET biosensors will be explored for the detection of hCG biomarkers in case vs control samples from patients. Furthermore, in the next stage of development of the GFETs, we will also investigate the specificity of the sensors, optimisation of the quality of CVD graphene, contact resistance and novel AC and RF detection techniques to enhance the sensitivity of the biosensors.

## Figures and Tables

**Table 1:** Summary of GFET resistance changes measured at different stages of the functionalisation process with 1 pg/mL to 1 ng/mL concentration of hCG antigen.

Stage	1 pg/mL (Ω)	ΔR (%)	1 ng/mL (Ω)	ΔR (%)
Bare	741.8	0	6686.3	0
Linker	2341.9	+215.7	3733.3	−44.2
Ab	2590.7	+10.6	3361.7	−11.1
BSA	3021.0	+16.6	4065.4	+20.9
hCG	6467.5	+114.1	16,468.5	+305.1
